# Malignancy risk stratification of thyroid nodules according to echotexture and degree of hypoechogenicity: a retrospective multicenter validation study

**DOI:** 10.1038/s41598-022-21204-5

**Published:** 2022-10-05

**Authors:** Ji Ye Lee, Chang Yoon Lee, Inpyeong Hwang, Sung-Hye You, Sun-Won Park, Boeun Lee, Ra Gyoung Yoon, Younghee Yim, Ji-hoon Kim, Dong Gyu Na

**Affiliations:** 1 Department of Radiology, Seoul National University Hospital, Seoul National University College of Medicine, Seoul, 03080 Korea; 2 Department of Radiology, Seoul National University College of Medicine, Seoul, 03080 Korea; 3 Department of Radiology, Research Institute and Hospital, National Cancer Center, Goyang, 10408 Republic of Korea; 4 Department of Radiology, Anam Hospital, Korea University College of Medicine, Seoul, 02841 Korea; 5 Department of Radiology, Seoul Metropolitan Government Seoul National University Boramae Medical Center, Seoul, 07061 Korea; 6 Department of Radiology, Ewha Womans University Seoul Hospital, Ewha Womans University, Seoul, 07804 Korea; 7 Department of Radiology, Nowon Eulji Medical Center, Eulji University College of Medicine, Seoul, 01830 Korea; 8 Department of Radiology, Chung-Ang University Hospital, Chung-Ang University College of Medicine, Seoul, 06973 Korea; 9 Department of Radiology, Gangneung Asan Hospital, University of Ulsan College of Medicine, Gangneung, 25440 Korea

**Keywords:** Endocrinology, Medical research

## Abstract

Various risk stratification systems show discrepancies in the ultrasound lexicon of nodule echotexture and hypoechogenicity. This study aimed to determine the malignancy risk of thyroid nodules according to their echotexture and degree of hypoechogenicity. From June to September 2015, we retrospectively evaluated 5601 thyroid nodules with final diagnoses from 26 institutions. Nodules were stratified according to the echotexture (homogeneous vs. heterogeneous) and degree of hypoechogenicity (mild, moderate, or marked). We calculated the malignancy risk according to composition and suspicious features. Heterogeneous hypoechoic nodules showed a significantly higher malignancy risk than heterogeneous isoechoic nodules (*P* ≤ 0.017), except in partially cystic nodules. Malignancy risks were not significantly different between homogeneous versus heterogeneous nodules in both hypoechoic (*P* ≥ 0.086) and iso- hyperechoic nodules (*P* ≥ 0.05). Heterogeneous iso-hyperechoic nodules without suspicious features showed a low malignancy risk. The malignancy risks of markedly and moderately hypoechoic nodules were not significantly different in all subgroups (*P* ≥ 0.48). Marked or moderately hypoechoic nodules showed a significantly higher risk than mild hypoechoic (*P* ≤ 0.016) nodules. The predominant echogenicity effectively stratifies the malignancy risk of nodules with heterogeneous echotexture. The degree of hypoechogenicity could be stratified as mild versus moderate to marked hypoechogenicity.

## Introduction

The echogenicity of a thyroid nodule on ultrasonography (US) is an important descriptor for distinguishing malignancy from benign nodules^1–4^. Previous studies have consistently reported that the malignancy risk of hypoechoic nodules was higher than that of iso- or hyperechoic nodules^1,3–5^. Marked hypoechogenicity is related to an increased risk of malignancy in thyroid nodules^1,5–8^ and has been adopted in several risk stratification systems (RSS)^9–12^.

Despite its importance, the US definition of nodule echogenicity shows discrepancies across risk stratification systems (RSS)^13^. For nodules with heterogeneous echogenicity, guidelines adopt different strategies; the Korean Thyroid Imaging Reporting and Data System (K-TIRADS) and American College of Radiology (ACR) TIRADS classified them based on the predominant echogenicity^11,14^, but the European Thyroid Imaging Reporting and Data System (EU-TIRADS) classified heterogeneous nodules as mildly hypoechoic nodules when they possessed any hypoechoic portion^10^. While the American Association of Clinical Endocrinologists/American College of Endocrinology/Associazione Medici Endocrinologi^9^, ACR^11^, EU-TIRADS^10^, and the Chinese Thyroid Imaging Reporting and Data System (C-TIRADS)^12^ distinguished between marked and mild hypoechogenicity, the American Thyroid Association^15^ and K-TIRADS^14^ did not for nodule risk stratification. In these RSSs including the 2016 K-TIRADS, nodules with a similar echogenicity to the anterior neck muscles (i.e., moderate hypoechogenicity) were grouped with nodules with mild hypoechogenicity^9–12,14,15^.

Our recent study demonstrated that nodule hypoechogenicity could be stratified as mild versus moderate to marked, and nodules with heterogeneous echogenicity are stratified by the predominant echogenicity of the solid portion^7^. According to the results of this study, the 2021 K-TIRADS revised the definition of marked hypoechogenicity as hypoechoic or similar echogenicity relative to the anterior neck muscles^16^. However, there is insufficient evidence on hypoechoic nodules’ stratification because the previous work involved a single-center^7^. We designed a multicenter study to determine if this revised definition of nodule hypoechogenicity could effectively stratify the malignancy risk of thyroid nodules. This study aimed to validate thyroid nodules’ malignancy risk according to their echotexture and degree of hypoechogenicity in a multicenter cohort.

## Results

### Demographic and clinicopathologic characteristics

The demographic data are summarized in Table 1. The mean size of the nodules was 53.4 ± 12.7 mm (range, 10–100 mm). Of the 5601 thyroid nodules, 4512 (80.6%) were diagnosed as benign and 1089 (19.4%) as malignant. The 1089 malignant nodules included 989 papillary thyroid carcinomas (90.8%), 62 follicular carcinomas (5.7%), 12 (1.1%) medullary carcinomas, 7 (0.6%) poorly differentiated carcinomas, 6 (0.6%) anaplastic carcinomas, 5 (0.5%) metastases, 4 (0.4%) unspecified malignancies, 3 (0.3%) lymphomas, and 1 (0.1%) squamous cell carcinoma. Patients with malignant nodules were significantly younger (*P* < 0.001), included a smaller proportions of female patients (*P* < 0.001), and smaller nodule size (*P* < 0.001) than patients with benign nodules.

**Table 1:** Demographic Data of 5601 Nodules in This Study.

Nodules	Total (N = 5601)	Benign (N = 4512)	Malignant (N = 1089)	*P*
Number of patients	4989	3991	998	
Age (years)	53.4 ± 12.7	54.6 ± 13.1	48.8 ± 13.9	< .001
No. of female patients (%)	4627 (82.6%)	3793 (84.1%)	834 (76.6%)	< .001
Mean maximal nodule size (mm)	20.7 ± 10.8	21.1 ± 10.7	19.1 ± 11.1	< .001
**US echotexture of nodules**				< .001
Homogeneous	4274 (76.3%)	3492 (77.4%)	782 (71.8%)	
Heterogeneous	1327 (23.7%)	1020 (22.6%)	307 (28.2%)	
**US echogenicity of nodules**^**a**^				< .001
Marked hypoechogenicity	315 (5.6%)	111 (2.5%)	204 (18.7%)	
Moderate hypoechogenicity	705 (12.6%)	365 (8.1%)	340 (31.2%)	
Mild hypoechogenicity	994 (17.7%)	760 (16.8%)	234 (21.5%)	
Iso- or hyperechogenicity	3587 (64.0%)	3276 (72.6%)	311 (28.6%)	

Unless otherwise indicated, data are numbers with percentages in parentheses for categorical variables.

^a^Determined by the predominant echogenicity in nodules with heterogeneous echotexture.

When we determined the heterogeneously echotextured nodules’ echogenicity by the predominant echogenicity, iso- or hyperechogenicity was the most common (64.0%) in all nodules. Among 778 hypoechoic malignant nodules, 718 (92.3%) were PTCs, including 58 (7.5%) follicular variant PTCs, and 30 (3.9%) were follicular carcinomas. In 311 iso- or hyperechoic malignant nodules, 271 (87.1%) were PTCs, including 69 (21.9%) follicular variant PTCs, and 32 (10.3%) were follicular carcinomas.

### Comparison of the malignancy risk between nodules with homogeneous and heterogeneous echotexture

Table 2 shows the malignancy risks of nodules classified according to their echogenicity and echotexture. Overall, the homogeneous hypoechoic nodules’ malignancy risk was significantly higher than heterogeneous hypoechoic nodules (40.5 vs. 33.5%, *P* = 0.022). Heterogeneous hypoechoic nodules showed significantly higher malignancy risk than heterogeneous iso- or hyperechoic nodules (33.5 vs. 15.8%, *P* < 0.001). Heterogeneous iso- or hyperechoic nodules showed a significantly higher malignancy risk than homogeneous iso- or hyperechoic nodules (15.8 vs. 6.7%, *P* < 0.001).

**Table 2:** Malignancy risk stratified by echogenicity and echotexture.

Echotexture	Echogenicity	All			With suspicious features^c^			No suspicious US features^c^		
		No of malignancy, N (%)	No. of nodules, N (%)	Malignancy risk (%)	No of malignancy, N (%)	No. of nodules, N (%)	Malignancy risk (%)	No of malignancy, N (%)	No. of nodules, N (%)	Malignancy risk (%)
**All**										
Homogeneous	Hypoechoic^b^	594 (54.5)	1465 (26.2)	40.5	448 (63.1)	686 (45.5)	65.3	146 (38.5)	779 (19.0)	18.7
	Iso- or hyperechoic	188 (17.3)	2809 (50.2)	6.7	66 (9.3)	371 (24.6)	17.8	122 (32.2)	2438 (59.6)	5.0
Heterogeneous^a^	Hypoechoic^b^	184 (16.9)	549 (9.8)	33.5	129 (18.2)	228 (15.1)	56.6	55 (14.5)	321 (7.8)	17.1
	Iso- or hyperechoic	123 (11.3)	778 (13.9)	15.8	67 (9.4)	223 (14.8)	30.0	56 (14.8)	555 (13.6)	10.1
Total		1089 (100.0)	5601 (100.0)	19.4	710 (100.0)	1508 (100.0)	47.1	379 (100.0)	4093 (100.0)	9.3
**Solid**										
Homogeneous	Hypoechoic^b^	554 (61.8)	1183 (38.9)	46.8	425 (67.6)	611 (56.6)	69.6	129 (48.1)	572 (29.2)	22.6
	Iso- or hyperechoic	110 (12.3)	1044 (34.3)	10.5	44 (7.0)	165 (15.3)	26.7	66 (24.6)	879 (44.8)	7.5
Heterogeneous^a^	Hypoechoic^b^	151 (16.8)	377 (12.4)	40.1	111 (17.6)	169 (15.6)	65.7	40 (14.9)	208 (10.6)	19.2
	Iso- or hyperechoic	82 (9.1)	437 (14.4)	18.8	49 (7.8)	135 (12.5)	36.3	33 (12.3)	302 (15.4)	10.9
Total		897 (100.0)	3041 (100.0)	29.5	629 (100.0)	1080 (100.0)	58.2	268 (100.0)	1961 (100.0)	13.7
**Partially cystic**										
Homogeneous	Hypoechoic^b^	40 (20.8)	282 (11.0)	14.2	23 (28.4)	75 (17.5)	30.7	17 (15.3)	207 (9.7)	8.2
	Iso- or hyperechoic	78 (40.6)	1765 (68.9)	4.4	22 (27.2)	206 (48.1)	10.7	56 (50.5)	1559 (73.1)	3.6
Heterogeneous^a^	Hypoechoic^b^	33 (17.2)	172 (6.7)	19.2	18 (22.2)	59 (13.8)	30.5	15 (13.5)	113 (5.3)	13.3
	Iso- or hyperechoic	41 (21.4)	341 (13.3)	12.0	18 (22.2)	88 (20.6)	20.5	23 (20.7)	253 (11.9)	9.1
Total		192 (100.0)	2560 (100.0)	7.5	81 (100.0)	428 (100.0)	18.9	111 (100.0)	2132 (100.0)	6.2

^a^Nodule echogenicity was categorized by the predominant echogenicity in nodules with heterogeneous echotexture.

^b^Hypoechogenicity includes any of marked-, moderate-, and mild hypoechogenicity.

^c^Suspicious US features include punctuate echogenic foci, nonparallel orientation (taller than wide) and irregular shape.

When we classified the nodules according to composition and the presence of suspicious features, there was no significant difference in malignancy risks between homogeneous hypoechoic and heterogeneous hypoechoic nodules in all subgroups (*P* ≥ 0.086). On the contrary, heterogeneous hypoechoic nodules showed significantly higher malignancy risks than heterogeneous isoechoic nodules in all subgroups (*P* ≤ 0.017) except partially cystic nodules. The malignancy risks were not significantly different between heterogeneous iso- or hyperechoic nodules and homogeneous isoechoic nodules in all subgroups except in the partially cystic nodules subgroup without suspicious features (*P* ≥ 0.05).

### Risk stratification of thyroid nodules with heterogeneous echotexture

In terms of risk stratification, the malignancy risks of solid heterogeneous hypoechoic nodules with suspicious features were stratified within the high suspicion category, along with solid homogeneous hypoechoic nodules with suspicious features (a malignancy risk of 65.7% in solid heterogeneous hypoechoic nodules with suspicious features and 69.6% in solid homogeneous hypoechoic nodules with suspicious features). The malignancy risks of solid heterogeneous iso- or hyperechoic nodules ranged within the intermediate suspicion category depending on the presence of suspicious US features (10.9% in nodules without suspicious features and 36.3% in nodules with suspicious features). The malignancy risk of solid homogeneous iso- or hyperechoic nodules ranged within the low-to-intermediate suspicion categories, depending on the presence of suspicious US features (7.5% in solid homogeneous iso- or hyperechoic nodules without suspicious features and 26.7% in solid homogeneous iso- or hyperechoic nodules with suspicious features).

In partially cystic nodules, the malignancy risks of hypoechoic or iso- or hyperechoic nodules (either homogeneous or heterogeneous) with suspicious features were stratified within the intermediate suspicion category (10.7–30.7%). The malignancy risks of all partially cystic nodules without suspicious features ranged within the low-to-intermediate suspicion category (3.6–13.3%), regardless of echogenicity and echotexture.

### Malignancy risk according to the degree of predominant hypoechogenicity

Table 3 lists the calculated malignancy risks of nodules categorized by their predominant degree of hypoechogenicity grouped by overall nodules and subgroups. Markedly hypoechoic nodules demonstrated a significantly higher malignancy risk than moderately (*P* < 0.001) and mildly hypoechoic (*P* < 0.001) nodules. The malignancy risks of markedly and moderately hypoechoic nodules were significantly higher than that of mildly hypoechoic nodules (*P* < 0.001).

**Table 3:** Malignancy Risk Stratified by Degree of Hypoechogenicity and Predominant Echogenicity According to Composition and suspicious Features.

Echogenicity	All			Nodules with suspicious features^a^			Nodules without suspicious features^a^		
	No. of malignant nodules, N (%)	No. of nodules, N (%)	Malignancy risk (%)	No. of malignant nodules, N (%)	No. of nodules, N (%)	Malignancy risk (%)	No. of malignant nodules, N (%)	No. of nodules, N (%)	Malignancy risk (%)
**All**									
Marked hypoechogenicity	204 (18.7)	315 (5.6)	64.8	178 (25.1)	228 (15.1)	78.1	26 (6.9)	87 (2.1)	29.9
Moderate hypoechogenicity	340 (31.2)	705 (12.6)	48.2	261 (36.8)	370 (24.5)	70.5	79 (20.8)	334 (8.2)	23.7
Mild hypoechogenicity	234 (21.5)	994 (17.7)	23.5	138 (19.4)	316 (21.1)	43.7	96 (25.3)	677 (16.6)	14.2
Iso- or hyperechogenicity	311 (28.6)	3587 (64.0)	8.7	133 (18.7)	594 (39.4)	22.4	177 (47.0)	2989 (73.1)	5.9
Total	1089 (100.0)	5601 (100.0)	19.4	710 (100.0)	1508 (100.0)	47.1	378 (100.0)	4087 (100.0)	9.2
**Solid**									
Marked hypoechogenicity	201 (22.4)	304 (10.0)	66.1	176 (28.0)	224 (20.7)	78.6	25 (9.3)	80 (4.1)	31.3
Moderate hypoechogenicity	312 (34.8)	596 (19.6)	52.3	244 (38.8)	333 (30.8)	73.3	68 (25.4)	263 (13.4)	25.9
Mild hypoechogenicity	192 (21.4)	660 (21.7)	29.1	116 (18.4)	223 (20.6)	52.0	76 (28.4)	437 (22.3)	17.4
Iso- or hyperechogenicity	192 (21.4)	1481 (48.7)	13.0	93 (14.8)	300 (27.8)	31.0	99 (36.9)	1181 (60.2)	8.4
Total	897 (100.0)	3041 (100.0)	29.5	629 (100.0)	1080 (100.0)	58.2	268 (100.0)	1961 (100.0)	13.7
**Partially cystic**									
Marked hypoechogenicity	3 (1.6)	11 (0.4)	27.3	2 (2.5)	4 (0.9)	50.0	1 (0.9)	7 (0.3)	14.3
Moderate hypoechogenicity	28 (14.6)	109 (4.3)	25.7	17 (21.0)	37 (8.6)	45.9	11 (9.9)	72 (3.4)	15.3
Mild hypoechogenicity	42 (21.9)	334 (13.0)	12.6	22 (27.2)	93 (21.7)	23.7	20 (18.0)	241 (11.3)	8.3
Iso- or hyperechogenicity	119 (62.0)	2106 (82.3)	5.7	40 (49.4)	294 (68.7)	13.6	79 (71.2)	1812 (85.0)	4.4
Total	192 (100.0)	2560 (100.0)	7.5	81 (100.0)	428 (100.0)	18.9	111 (100.0)	2132 (100.0)	5.2

^a^Suspicious US features include punctuate echogenic foci, nonparallel orientation (taller than wide) and irregular shape.

When we categorized nodules according to composition and presence of suspicious features, there was no significant difference in malignancy risk between markedly and moderately hypoechoic nodules in all subgroups, regardless of composition and the presence of suspicious features (*P* ≥ 0.48). In solid nodules, markedly or moderately hypoechoic nodules showed a significantly higher malignancy risk than mild hypoechoic (*P* ≤ 0.016) and iso- or hyperechoic (*P* < 0.001) nodules, regardless of suspicious features.

In partially cystic nodules with suspicious features, moderately hypoechoic nodules showed significantly higher malignancy risk than mild hypoechoic (*P* ≤ 0.045) and iso- or hyperechoic nodules (*P* < 0.001). There was no significant difference in malignancy risk in partially cystic nodules without suspicious features according to the degree of hypoechogenicity (*P* ≥ 0.116). Moderately (*P* = 0.008) and mildly hypoechoic (*P* = 0.017) nodules showed significantly higher malignancy risk than iso- or hyperechoic nodules in partially cystic nodules without suspicious features.

### Risk stratification of thyroid nodules according to the degree of hypoechogenicity

In solid nodules, the malignancy risks of nodules with moderate (73.3%) or marked hypoechogenicity (78.6%) with suspicious features were within the high suspicion category. Solid nodules with mild hypoechogenicity and suspicious features showed a slightly lower malignancy risk than the lower margin of the high suspicion category (52.0%). Solid, marked (31.3%), moderate (25.9%), and mild hypoechoic nodules (17.4%) without suspicious features were stratified as intermediate risk.

In partially cystic nodules, marked (50.0%) and moderate (45.9%) hypoechogenicity showed slightly higher malignancy risks than the estimated range of the intermediate and low suspicion categories according to the presence of suspicious features. Partially cystic nodules with mild hypoechogenicity and iso- or hyperechogenicity were classified within the low and intermediate suspicion categories according to the presence of suspicious features.

### Comparisons of malignancy risks among four nodule groups, based on composition and echogenicity

Table 4 illustrates the malignancy risks in the four groups of nodules categorized according to a combination of composition, predominant echogenicity, and presence of suspicious features. The malignancy risks differed significantly between these groups in the following decreasing order: solid hypoechoic, partially cystic hypoechoic, solid iso- or hyperechoic, and partially cystic iso- or hyperechoic. The malignancy risks significantly differed in all subgroups (all, *P* < 0.001) except between partially cystic hypoechoic versus solid iso- or hyperechoic nodules (*P* ≥ 0.122), regardless of the presence of suspicious features.

**Table 4:** Malignancy risk of four nodule categories based on composition and predominant echogenicity.

	Solid hypoechoic		Partially cystic hypoechoic		Solid isoechoic		Partially cystic isoechoic	
	No. of malignant nodules/all	Malignancy risk (95% CI)	No. of malignant nodules/all	Malignancy risk (95% CI)	No. of malignant nodules/all	Malignancy risk (95% CI)	No. of malignant nodules/all	Malignancy risk (95% CI)
All	705/1560	45.2 (41.9, 48.7)	73/454	16.1 (12.6, 20.2)	192/1481	13.0 (11.2, 14.9)	119/2106	5.7 (4.7, 6.8)
*P*	< .001^a^		0.122^b^				< .001^c^	
Any suspicious feature	536/780	68.7 (63.0, 74.8)	41/134	30.6 (22.0, 41.5)	93/300	31.0 (25.0, 38.0)	40/294	13.6 (9.7, 18.5)
*P*	< .001^a^		0.954^b^				< .001^c^	
No suspicious feature	169/780	21.7 (18.5, 25.2)	32/320	10.0 (6.8, 14.1)	99/1181	8.4 (6.8, 10.2)	79/1812	4.4 (3.5, 5.4)
*P*	< .001^a^		0.385^b^				< .001^c^	

^a^Solid hypoechoic versus partially cystic hypoechoic.

^b^Partially cystic hypoechoic versus Solid isoechoic.

^c^Solid isoechoic versus partially cystic isoechoic.

### Reproducibility of nodule echotexture and hypoechogenicity

The four categories of iso- or hyperechoic and mild, moderate, and marked nodule hypoechogenicity had a substantial agreement (k = 0.79, 95% CI 0.76, 0.82). The four categories combined with nodule echotexture and echogenicity of homogeneous hypoechoic, heterogeneous hypoechoic, heterogeneous isohyperechoic, and homogeneous isohyperechogenicity also showed a substantial agreement (k = 0.77, 0.75, 0.81).

## Discussion

Our study demonstrated no significant difference in malignancy risks between homogeneous vs. heterogeneous hypoechoic nodules in all subgroups and homogenous vs. heterogeneous iso- or hyperechoic nodules in all subgroups except partially cystic nodules without suspicious features. Heterogeneous hypoechoic nodules showed significantly higher malignancy risk than heterogeneous isoechoic nodules in all subgroups except partially cystic nodules. Our study validated that classifying nodules by their predominant echogenicity is a reasonable form of risk stratification. Regarding the degree of hypoechogenicity, nodules with moderate hypoechogenicity showed similar malignancy risks compared to markedly hypoechoic nodules. In contrast, moderately hypoechoic nodules showed significantly higher malignancy risks than mild hypoechoic nodules in all subgroups except partially cystic nodules without suspicious features. Based on our results, moderately hypoechoic nodules should be grouped with marked hypoechoic nodules for risk stratification.

For nodules with heterogeneous echogenicity, the EU-TIRADS suggested that nodules with any hypoechoic component should be regarded as hypoechoic nodules and classified as intermediate risk^10^. However, in our study, the malignancy risks of heterogeneous isoechoic nodules were not significantly different from their homogeneous counterparts except in the partially cystic nodules without suspicious features subgroup. This result aligns with the findings of our previous study^7^. Although the malignancy risks of heterogeneous isoechoic nodules were higher than homogeneous isoechoic nodules and overall nodules, the malignancy risks of heterogeneous isoechoic nodules ranged within the low-to-intermediate risk categories, depending on concurrent suspicious US features. Therefore, our study’s results support the strategy provided by K-TIRADS and ACR TIRADS for assessing nodules with heterogeneous echogenicity.

In the EU-TIRADS^10^ and ACR-TIRADS^11^, moderate hypoechogenicity was classified as a similar risk to mild hypochogenicity. However, in this study, moderate hypochogenicity showed a similar malignancy risk to marked hypochogenicity. The results of this study suggest that the previous definition of marked hypoechogenicity should be revised as hypoechoic or similar echogenicity relative to the anterior neck muscles. The result of this study confirmed the validity of the revised definition of marked hypoechogenicity by 2021 K-TIRADS.

Our results are in line with those of our previous study in that the malignancy risks of moderately hypoechoic nodules are similar to that of markedly hypoechoic nodules^7^. However, the results in overall nodules were somewhat discrepant from the previous study^7^, demonstrating that the malignancy risks of marked hypoechoic nodules were higher than that of moderately hypoechoic nodules^6^. In this cohort, concurrent suspicious US features occurred more frequently in marked hypoechoic nodules than moderately hypoechoic nodules (marked hypoechoic, 72.4% vs. moderate hypoechoic, 52.5%, *P* < 0.001). The higher prevalence of suspicious features in marked hypoechoic nodules might have caused confounding effects in the malignancy risks between these two groups.

In the partially cystic nodules without suspicious features subgroup, the malignancy risks of most nodules ranged within the low-risk category, regardless of their predominant echogenicity. This contrasts partially cystic nodules with suspicious features’ risks, which fell within the intermediate-risk category in most nodules. In contrast to solid nodules, partially cystic nodules without suspicious features showed no significant difference in malignancy risk between marked/moderate versus mild hypoechoic nodules, and the difference between various degrees of hypoechogenicity was diminished. Additionally, in partially cystic nodules, most partially cystic hypoechoic nodules showed mild hypoechogenicity (68.7–85.0%) and the incidence of marked hypoechogenicity was very rare. We assume that in partially cystic nodules, malignancy risk is mainly determined by the presence of suspicious features and the degree of hypoechogenicity had little impact.

Our study has several limitations. First, the reference standards for some benign nodules were based on one biopsy result, which may cause false-negative results. However, considering that the majority of false-negative rates (FNR) occur in high suspicion nodules^17^, the rate of FNR might be negligible in this study (simulated false-negative rate in nodules with one benign biopsy result 1.4%). Second, we retrospectively assessed the nodules’ US features, which may limit the accuracy of interpretation. Regarding echogenicity, subtle echo changes could be misclassified in prerecorded US images. Moreover, focal suspicious features could be missed when archiving images during dynamic exploration and these points could potentially impact nodule categorization. However, a recent study reported that overall inter-exam agreement between real-time and retrospective US image interpretation for thyroid nodules was more than substantial in 2016 K-TIRADS^18^. Although this method might be suboptimal, we speculate that retrospective evaluation of US images could be an alternative assessment method of malignancy risk in thyroid nodules. Third, although our study demonstrated this proposed classification of echogenicity showed good reproducibility, determination of US lexicons can be still affected by interobserver variability^19^. In this context, artificial intelligence techniques might have a potential complementary role^20,21^. Future in-depth studies are needed to validate the reproducibility of this US lexicon in multiple readers. The malignancy rate of this cohort (19.4%) is relatively high considering the prevalence of malignancy among all thyroid nodules. We speculate that this is because this cohort was mainly consisted of biopsy required nodules, and the majority of institutions were tertiary referral hospitals. Regarding a recent meta-analysis, the average malignancy rate of published thyroid nodule cohorts was 27.8 ± 15.3 (range 3.9–56.2)^22^. Therefore, we assume that the malignancy rate in this cohort appears acceptable considering the specific aim of this study.

In conclusion, the malignancy risk of nodules with heterogeneous echotexture can be stratified based on predominant echogenicity. Additionally, nodule hypoechogenicity can be classified as mild vs. moderate to marked hypoechogenicity for malignancy risk stratification.

## Materials and methods

The institutional review boards (IRB) of the 26 participating centers (CHA Gangnam Medical Center, Chung-Ang University Hospital, Konkuk University Medical Center, Gyeongsang National University Hospital, Korea University Anam Hospital, Kosin University Gospel Hospital, Daejeon St. Mary’s Hospital, Dongguk University Ilsan Hospital, Seoul National University Hospital, Seoul Metropolitan Government Seoul National University Boramae Medical Center, Soonchunhyang University Seoul Hospital, Nowon Eulji Medical Center, Busan Paik Hospital, Inje University Haeundae Paik Hospital, Hanyang University Guri Hospital, Asan Medical Center, Ajou University Hospital, GangNeung Asan Hospital, Korea University Ansan Hospital, Seoul National University Bundang hospital, National Cancer Center, Soonchunhyang University Bucheon Hospital, Gangwon University Hospital, Chonnam National University Hwasun Hospital, Gachon University Gil Medical Center and Seoul St. Mary’s Hospital) approved this study. This study was conducted in accordance with the Declaration of Helsinki. The informed consent requirement was waived for this retrospective review from the IRB of participating centers.

### Study population

**Figure 1:**
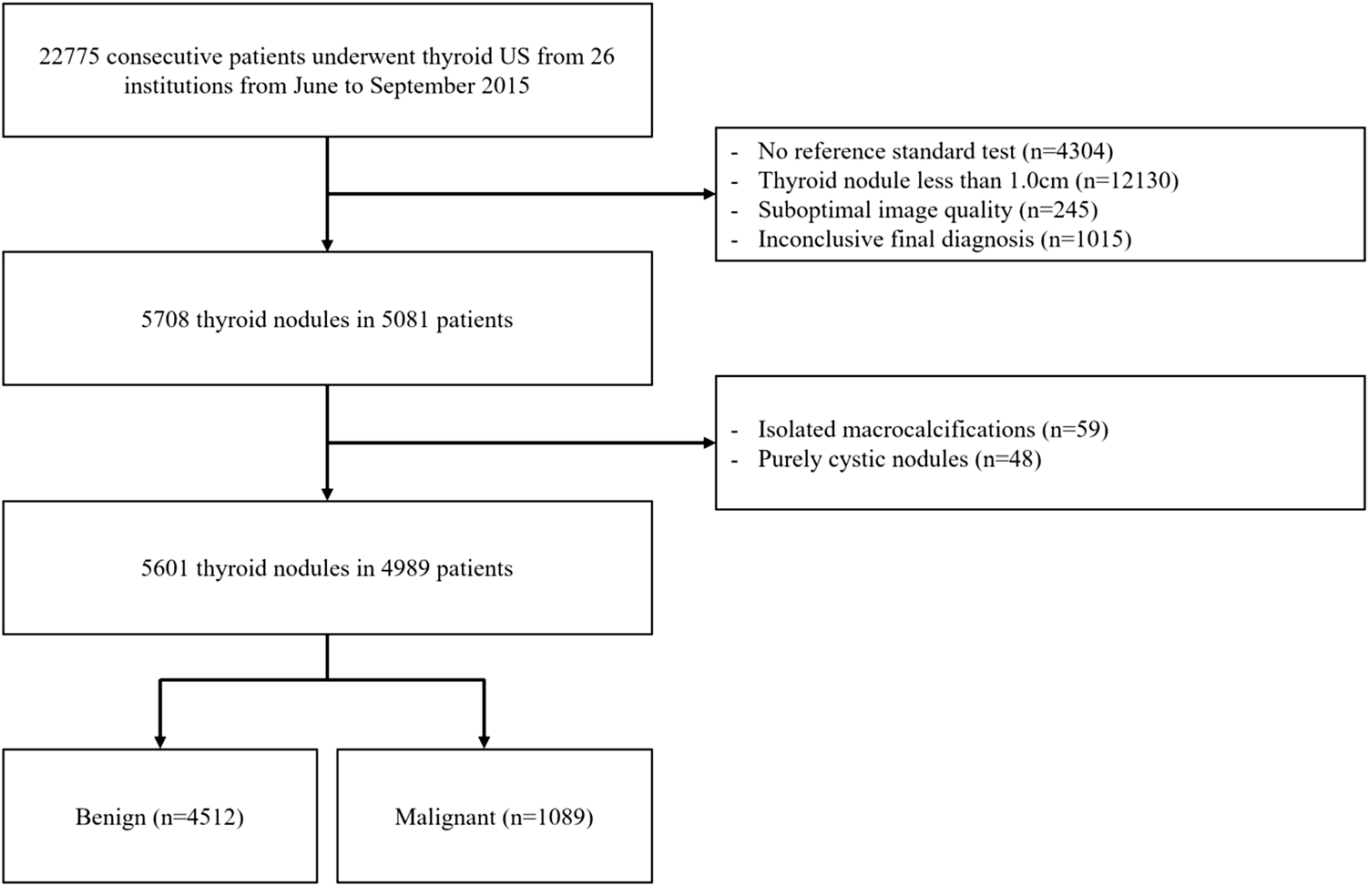
Flowchart of the study. US = ultrasonography, numbers are nodules numbers, unless otherwise specified.

Patient data were retrospectively collected from 26 different hospitals in Korea (Thyroid Imaging Network of Korea registry, THINK). From June to September 2015, 22,775 patients underwent thyroid US at the 26 participating institutions. Among them, 16,679 patients were excluded due to a lack of reference standard test (biopsy or surgery) (n = 4304), thyroid nodules < 1.0 cm (maximal diameter, n = 12,130), suboptimal image quality (n = 245), or inconclusive/indeterminate biopsy results (Bethesda I; nondiagnostic or unsatisfactory or III; atypia of undetermined significance or follicular lesion of undetermined significance, n = 1015 patients with 1102 nodules)^23,24^. In this study, 59 isolated macrocalcifications and 48 purely cystic nodules were further excluded because of an inability to assess nodule echogenicity. We included a total of 5,601 thyroid nodules in 4989 patients in this study (4101 women, 888 men; age range: 19–76 years) (Fig. 1). Malignant nodules were diagnosed based on the histopathological results after surgery (n = 927) or malignant (Bethesda VI) fine needle aspiration (FNA) or core-needle biopsy (CNB) results (n = 162). Benign nodules were diagnosed based on the histopathological results after surgery (n = 390), with at least two benign FNA or CNB results (n = 594) and one benign FNA or CNB result (n = 3528). All cases with a preoperative diagnosis on Bethesda V with a diagnosis of malignancy were confirmed as thyroid cancer on surgical biopsy.

### US examination and image analysis

**Figure 2:**
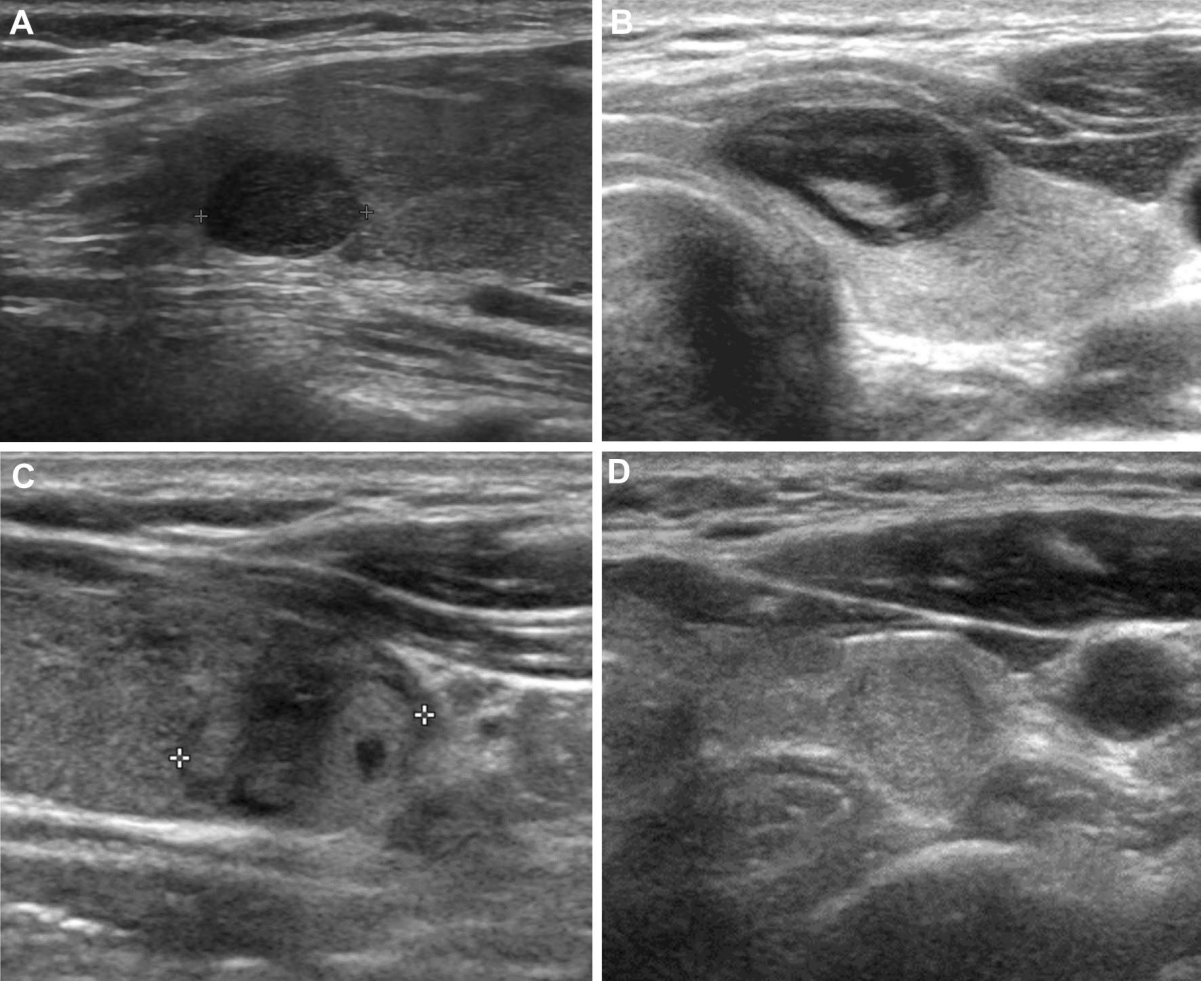
Thyroid nodules classified according to the echotexture and echogenicity. (**A**) A nodule with homogeneous hypoechogenicity. Diagnosis: Conventional papillary thyroid carcinoma (**B**) A nodule with heterogeneous, predominant hypoechogenicity. Note internal iso- or hyperechoic solid portions consisting less than 50% of the nodule. Diagnosis: Conventional papillary thyroid carcinoma. (**C**) Nodule with heterogeneous, predominant iso- or hyperechogenicity. The hypoechoic solid portion accounts for less than 50% of the nodule. Diagnosis: Benign follicular nodule in core needle biopsy. D. Nodule with homogeneous iso- or hyperechogenicity. Diagnosis: Benign follicular nodule in core needle biopsy.

All US examinations were performed with a 10–14 MHz linear probe. US images were retrospectively reviewed by one of 17 experienced radiologists with 8–22 years of experience performing thyroid US using an online program (AIM AiCRO; http://study.aim-aicro.com). As 26 hospitals participated in this study, we were not able to put the names of all institutions’ US units. Before the multicenter study began, we held training sessions to establish a baseline consensus regarding US criteria^9–11,14,23,24^. The 17 radiologists evaluated images of biopsy-proven masses not included in the study and were asked to assess the US criteria during a consensus meeting, including composition, echogenicity, margin, calcification, orientation (taller-than-wide), spongiform appearance, and intracystic echogenic foci with comet tail artifact. All of the reviewers, who were blind to the FNA results and final diagnoses, then assessed the US features of the thyroid nodules. Reviewers determined the nodule hypoechogenicity by assessing the echogenicity of the solid component in a nodule. In the 2021 K-TIRADS, the nodule was defined as hypoechoic if it was hypoechoic relative to the normal thyroid parenchyma. The echotexture of the nodule was categorized as having homogenous or heterogeneous echotexture based on the uniformity of the nodule echogenicity^7^ (Fig. 2). Heterogeneous echotexture was defined as the nodule’s solid component showing two different portions of echogenicity (iso- or hyperechoic vs. hypoechogenicity). Heterogeneously echotextured nodules’ echogenicity was determined by their predominant echogenicity. The nodules were classified into four groups: homogeneous hypoechoic, heterogeneous hypoechoic, heterogeneous iso- or hyperechoic, and homogeneous iso- or hyperechoic.

**Figure 3:**
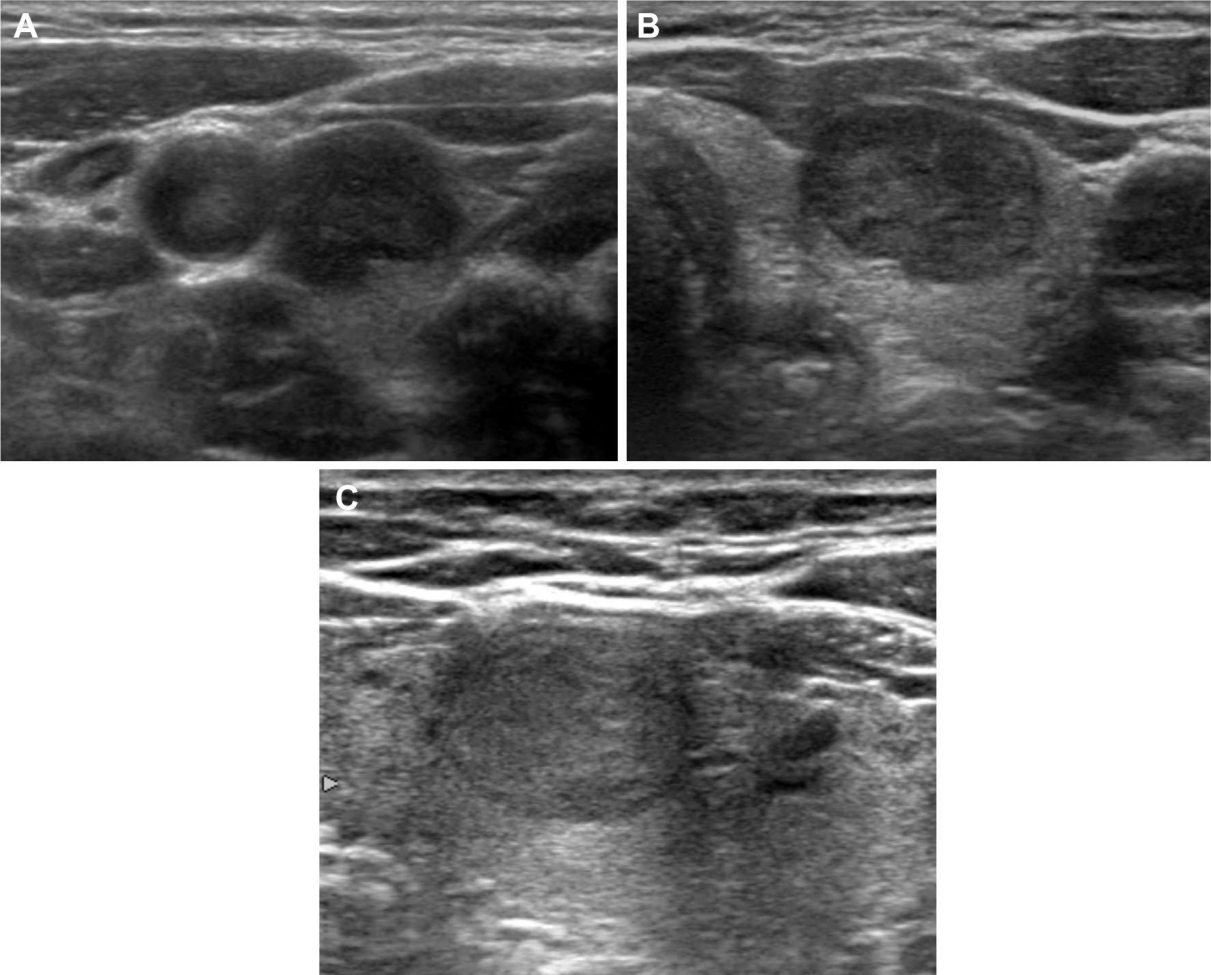
Hypoechoic thyroid nodules with various degree of hypoechogenicity. (**A**) Markedly hypoechoic nodule (hypoechoic relative to the anterior neck muscles) Diagnosis: Conventional papillary thyroid carcinoma (**B**) Moderately hypoechoic nodule (similar echogenicity to the anterior neck muscle). Diagnosis: Conventional papillary thyroid carcinoma (**C**) Mildly hypoechoic nodule (hypoechoic relative to the normal thyroid parenchyma, but hyperechoic relative to the anterior neck muscle). Diagnosis: Follicular variant papillary thyroid carcinoma.

The degree of hypoechogenicity was categorized as mild (hypoechoic relative to the thyroid parenchyma, but hyperechoic relative to the anterior neck muscles), moderate (similar echogenicity to the anterior neck muscles), and marked (hypoechoic relative to the anterior neck muscles) (Fig. 3)^5,7^. We assessed other US characteristics regarding the composition and presence of suspicious features (punctuate echogenic foci, nonparallel orientation, and irregular margin) of the thyroid nodules based on the 2021 K-TIRADS^16^. In the 2021 K-TIRADS, punctate echogenic foci were defined as punctate (≤ 1 mm) hyperechoic foci within the solid component of a nodule, nonparallel orientation as the anteroposterior diameter of a nodule being longer than its transverse diameter in the transverse plane, and irregular margin as a non-smooth edge with spiculation or microlobulation. The suggested malignancy risk in the 2021 K-TIRADS is as follows^16^: high suspicion, > 60%; intermediate suspicion, 10–40%; low suspicion 3–0%; and benign, < 3%.

The interobserver agreement on nodule echogenicity and echotexture was assessed on 1400 (25%) of 5601 nodules by another radiologist (J.Y.L, with eight years of experience in thyroid imaging). This reader randomly selected and blindly assessed the nodules.

### Data analysis and statistical analysis

We calculated each nodule category’s frequency and malignancy risk based on its echotexture and degree of hypoechogenicity. Chi-squared or Fisher’s exact tests were used to compare the malignancy risk among each group. We performed a subgroup analysis to assess the difference between the malignancy risks of thyroid nodules according to their composition (solid vs. partially cystic), and the presence of any suspicious US features. This reflected that the thyroid nodules’ malignancy risks differ according to their composition, echogenicity, and presence of suspicious US features^4^. Chi-squared or Fisher’s exact tests were used to compare the malignancy risk between homogeneous hypoechoic, heterogeneous hypoechoic, heterogeneous iso- or hyperechoic nodules, and homogeneous iso- or hyperechoic nodules. The interobserver agreement for the degree of echogenicity and echotexture was calculated using the Cohen κ statistic. All κ values were interpreted as follows: 0–0.20, slight; 0.21–0.40, fair; 0.41–0.60, moderate; 0.61–0.80, substantial; and 0.81–1.0, almost perfect agreement^25^. Statistical analyses were performed using IBM SPSS Statistics for Windows, Version 24.0 (IBM Corporation, Armonk, NY, USA, https://www.ibm.com/kr-ko/analytics/spss-statistics-software), and MedCalc (version 20.009, MedCalc Software Ltd, Ostend, Belgium, https://www.medcalc.org; 2022). A *P* value < 0.05 was considered statistically significant.

### Ethics approval

This retrospective study was approved by the institutional review boards of 26 participating centers.

### Consent to participate

The requirement for patient informed consent was waived.

## Data Availability

The datasets analyzed in the current study are available from the corresponding author on reasonable request.
